# Intake of compound probiotics accelerates the construction of immune function and gut microbiome in Holstein calves

**DOI:** 10.1128/spectrum.01909-23

**Published:** 2024-04-23

**Authors:** Xinghua Cai, Ping Yi, Xuewen Chen, Junhua Wu, Ganqiu Lan, Shijian Li, Shasha Luo, Fengdie Huang, Jinrong Huang, Peihong Shen

**Affiliations:** 1 State Key Laboratory for Conservation and Utilization of Subtropical Agro-bioresources, Guangxi Research Center for Microbial and Enzyme Engineering Technology, College of Life Science and Technology, Guangxi University, Nanning, Guangxi, China; 2 National Engineering Research Center for Non-Food Biorefinery, State Key Laboratory of Non-Food Biomass and Enzyme Technology, Guangxi Key Laboratory of Bio-refinery, Guangxi Research Center for Biological Science and Technology, Guangxi Academy of Sciences, Nanning, Guangxi, China; 3 College of Animal Science and Technology, Guangxi University, Nanning, Guangxi, China; 4 Guangxi UBIT Biotechnology Co., Ltd., Nanning, China; Beijing Institute of Genomics, Chinese Academy of Sciences, Beijing, China

**Keywords:** Holstein calves, gut microbiome, compound probiotics, metagenome sequencing, immune function

## Abstract

Acquired immunity is an important way to construct the intestinal immune barrier in mammals, which is almost dependent on suckling. To develop a new strategy for accelerating the construction of gut microbiome, newborn Holstein calves were continuously fed with 40 mL of compound probiotics (containing *Lactobacillus plantarum* T-14, *Enterococcus faecium* T-11, *Saccharomyces cerevisiae* T-209, and *Bacillus licheniformis* T-231) per day for 60 days. Through diarrhea rate monitoring, immune index testing, antioxidant capacity detection, and metagenome sequencing, the changes in diarrhea incidence, average daily gain, immune index, and gut microbiome of newborn calves within 60 days were investigated. Results indicated that feeding the compound probiotics reduced the average diarrhea rate of calves by 42.90%, increased the average daily gain by 43.40%, raised the antioxidant indexes of catalase, superoxide dismutase, total antioxidant capacity, and Glutathione peroxidase by 22.81%, 6.49%, 8.33%, and 13.67%, respectively, and increased the immune indexes of IgA, IgG, and IgM by 10.44%, 4.85%, and 6.12%, respectively. Moreover, metagenome sequencing data showed that feeding the compound probiotics increased the abundance of beneficial strains (e.g., *Lactococcus lactis* and *Bacillus massionigeriensis*) and decreased the abundance of some harmful strains (e.g., *Escherichia sp. MOD1-EC5189* and *Mycobacterium brisbane*) in the gut microbiome of calves, thus contributing to accelerating the construction of healthy gut microbiome in newborn Holstein calves.

**IMPORTANCE:**

The unstable gut microbiome and incomplete intestinal function of newborn calves are important factors for the high incidence of early diarrhea. This study presents an effective strategy to improve the overall immunity and gut microbiome in calves and provides new insights into the application of compound probiotics in mammals.

## INTRODUCTION

The composition and function of the gut microbiome are key factors that affect the intestinal health of mammals and play an extremely important role in promoting animal growth and development, maintaining the stability of the microenvironment *in vivo*, and regulating nerve excitement (1–3). For most mammals, a stable gut microbiome can effectively increase digestion and absorption of nutrients, accelerate the construction of immune function of intestinal epithelial cells, promote the development of the immune system, and inhibit the growth of pathogenic microorganisms, thus contributing to reducing the occurrence of diseases (4, 5). During the infancy of mammals, the diversity and composition of the gut microbiome are easily influenced by diet, antibiotic intake, pathogen infection, and other factors (6–8), which leads to a series of clinical diseases, such as malnutrition, low immunity, and high diarrhea rate. This further highlights the importance of establishing early gut microbiome in mammals. However, almost all mammals rely on suckling in infancy to gradually form a stable gut microbiome, which takes a relatively long time. Therefore, it is urgent to explore effective strategies to accelerate the construction of gut microbiome in infancy.

As a kind of important mammal, researches on the composition and function of gut microbiome in Holstein cows have attracted wide attention. However, there is still a lack of systematic understanding on how to rapidly improve the intestinal function of Holstein calves and establish a stable gut microbiome (9–11). Generally, Holstein calves are prone to problems such as intestinal dysfunction, low immunity, malabsorption, and relatively slow growth within 30 days of age and usually need antibiotic treatment or intervention by changing feeding methods (12, 13). For example, the National Animal Health Monitoring System reported that diarrhea is the most common disease affecting calves: 21% of calves were diagnosed with intestinal diseases, and 76% of these calves were treated with antibiotics (14). Studies have also indicated that early feeding fermented feed can improve the antioxidant capacity, growth performance, food intake, and health status of calves, reduce pro-inflammatory cytokines, and alleviate weaning stress (15, 16). In addition, direct feeding of specific probiotics is beneficial to increase the average daily gain of calves, improve intestinal microbial homeostasis, and feed digestibility (17). However, different probiotics or fermented feed may produce inconsistent results. Compared with feeding a single probiotic or fermented feed, compound probiotics have shown better effects in treating antibiotic-associated diarrhea (18, 19), resisting bacterial infection, and regulating the structure of the gut microbiome (20, 21), which indicates that compound probiotics may have greater potential applications.

Although probiotics have been widely used in animal or human health, their regulation mechanism is still unclear and needs further study. In general, the function of probiotics is considered to be closely related to its metabolites, which are beneficial to improve the intestinal microenvironment by producing a variety of short-chain fatty acids (e.g., butyrate, propionate, etc.) (22). In addition, probiotics can also produce antimicrobial compounds (e.g., bacteriocins) to inhibit the growth of pathogenic bacteria or directly act by regulating the endogenous microflora (23, 24). Up to now, it is well known that probiotics can regulate gut microbiome by enhancing epithelial defense or directly competing with pathogenic microorganisms for mucosal adhesion sites, thus affecting the intestinal barrier function and intestinal health of the body (25, 26). However, a large number of studies have focused on the effects of feeding probiotics on poultry health, but less attention has been paid to the influences of compound probiotics on the immune function and gut microbiome of newborn calves, and its regulation mechanism is still unclear. Therefore, it is of great theoretical and practical significance to explore the effects of compound probiotics on immune function and the construction of intestinal microbiota in newborn Holstein calves.

Previous experiments have found that feeding compound probiotics can significantly reduce the diarrhea rate of newborn calves and help to improving daily weight gain and overall health. Based on the important findings, it was hypothesized that the intake of compound probiotics could effectively regulate the gut microbiome of calves, improve their intestinal function and overall immunity, and accelerate the construction of the immune barrier in newborn calves. To verify these speculations, metagenome sequencing, antioxidant capacity detection, and immune index detection were carried out to systematically explore the effects of compound probiotics on Holstein calves. This work provides new insights for the research and application of compound probiotics in regulating the gut microbiome and overall immune function of animals.

## RESULTS

### Quality evaluation of the compound probiotics

**Fig 1:**
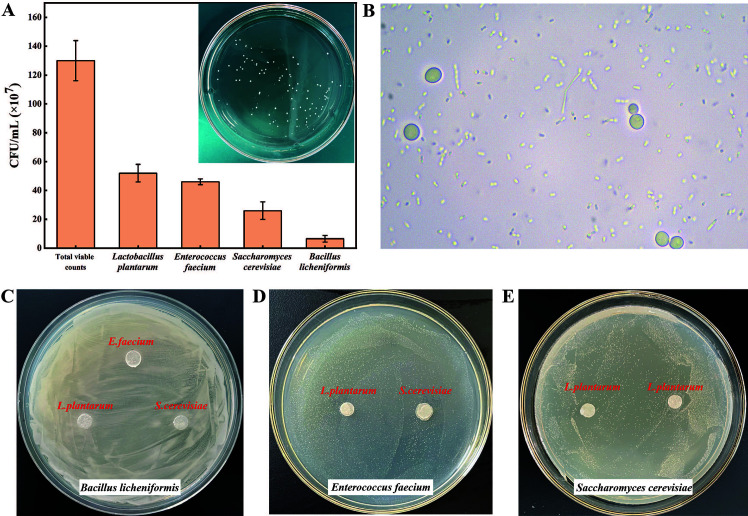
Quality evaluation of the compound probiotics. (**A**) The viable cells of the compound probiotics. (**B**) The viable cells of compound probiotics under a microscope. (**C**) Biological antagonism test of *B. licheniformis T-231* against *L. plantarum T-14*, *S. cerevisiae* T-209, and *E. faecium* T-11. (**D**) Biological antagonism test of *E. faecium* T-11 against *L. plantarum* T-14 and *S. cerevisiae* T-209. (**E**) Biological antagonism test of *S. cerevisiae* T-209 against *L. plantarum* T-14.

To verify whether the prepared compound probiotic reached the expected dose level of more than 10^8^ colony-forming units (CFU)/mL, its viable counts were detected by plate counting method. As shown in Fig. 1, the total viable counts of the compound probiotics were 1.3 × 10^9^ CFU/mL. Among which the viable counts of *Bacillus licheniformis* T-231 was 6.5 × 10^7^ CFU/mL, *Saccharomyces cerevisiae* T-209 was 2.6 × 10^8^ CFU/mL, *Enterococcus faecium* T-11 was 4.6 × 10^8^ CFU/mL, and *Lactobacillus plantarum* T-14 was 5.2 × 10^8^ CFU/mL, respectively (Fig. 1A). In addition, these four isolated probiotic strains could coexist in a fermentation system without antagonistic effect (Fig. 1B through E), but their viable counts were not equal though they were compounded in the same proportion. This could be due to the initial concentration of bacteria being inconsistent or their growth rates being different. However, the prepared compound probiotics have the expected high viable counts of more than 10^8^ CFU/mL, which could be used in the follow-up feeding experiment of newborn Holstein calves.

### Feeding compound probiotics reduced diarrhea rate and improved the growth performance of Holstein calves

**Fig 2:**
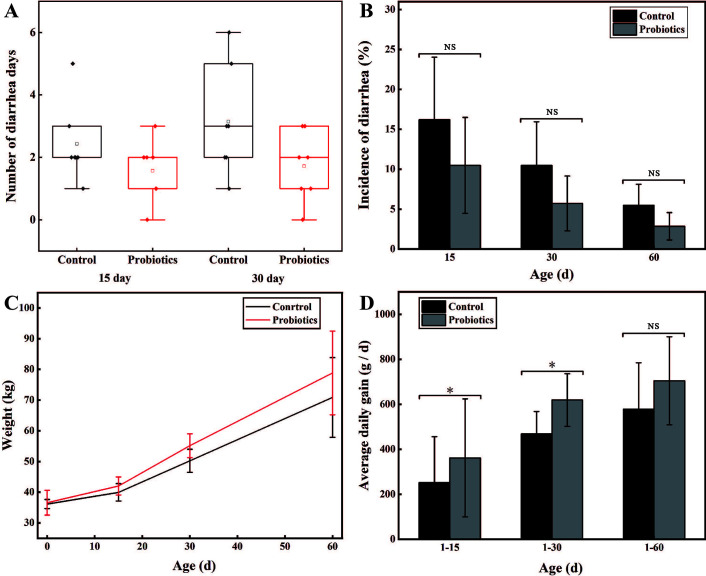
Effects of feeding compound probiotics on the newborn calves. (**A**) Diarrhea of calves within 60 days old. (**B**) Comparison of diarrhea rates of calves within 60 days. (**C**) Weight gain of calves within 60 days old. (**D**) Comparison of average daily gain of calves within 60 days. Note: “*” means “*P* < 0.05” and “NS” refers to “Non-significant.”

The effects of the compound probiotics on diarrhea rate and growth performance of calves were studied to verify its potential application. As shown in Fig. 2, within 15 days old, the average diarrhea rate of calves fed with compound probiotics was 10.48%, while that of control was 16.19% (*P* > 0.05). Moreover, none of the calves fed with compound probiotics developed diarrhea after 17 days, while the control group still developed diarrhea within 35 days old (Fig. 2A). Overall, the average diarrhea rate of calves fed with compound probiotics was 5.71% within 30 days, while that of calves fed with normal saline is 10.48% (*P* > 0.05; Fig. 2B), which indicated that feeding the compound probiotics could reduce diarrhea rate of newborn calves.

Furthermore, the calves fed with probiotics showed a better growth rate. At the age of 15 days, the average daily weight gain rate of calves fed with probiotics was 15.71%, while that of the control group was 10.61% (*P* < 0.05; Fig. 2C). At the age of 30 days, the average daily gain of calves fed with probiotics was significantly different from that fed with normal saline (*P* < 0.05). In addition, at the age of 60 days, the average daily gain of calves fed with probiotics was 704.76 g/day, which was 21.81% higher than that of the control (578.57 g/day; Fig. 2D). These results showed that feeding the compound probiotics is helpful to improve the average daily weight gain of calves and accelerating the growth rate of newborn calves.

### Feeding compound probiotics contributed to increasing the antioxidant ability of calves

**Fig 3:**
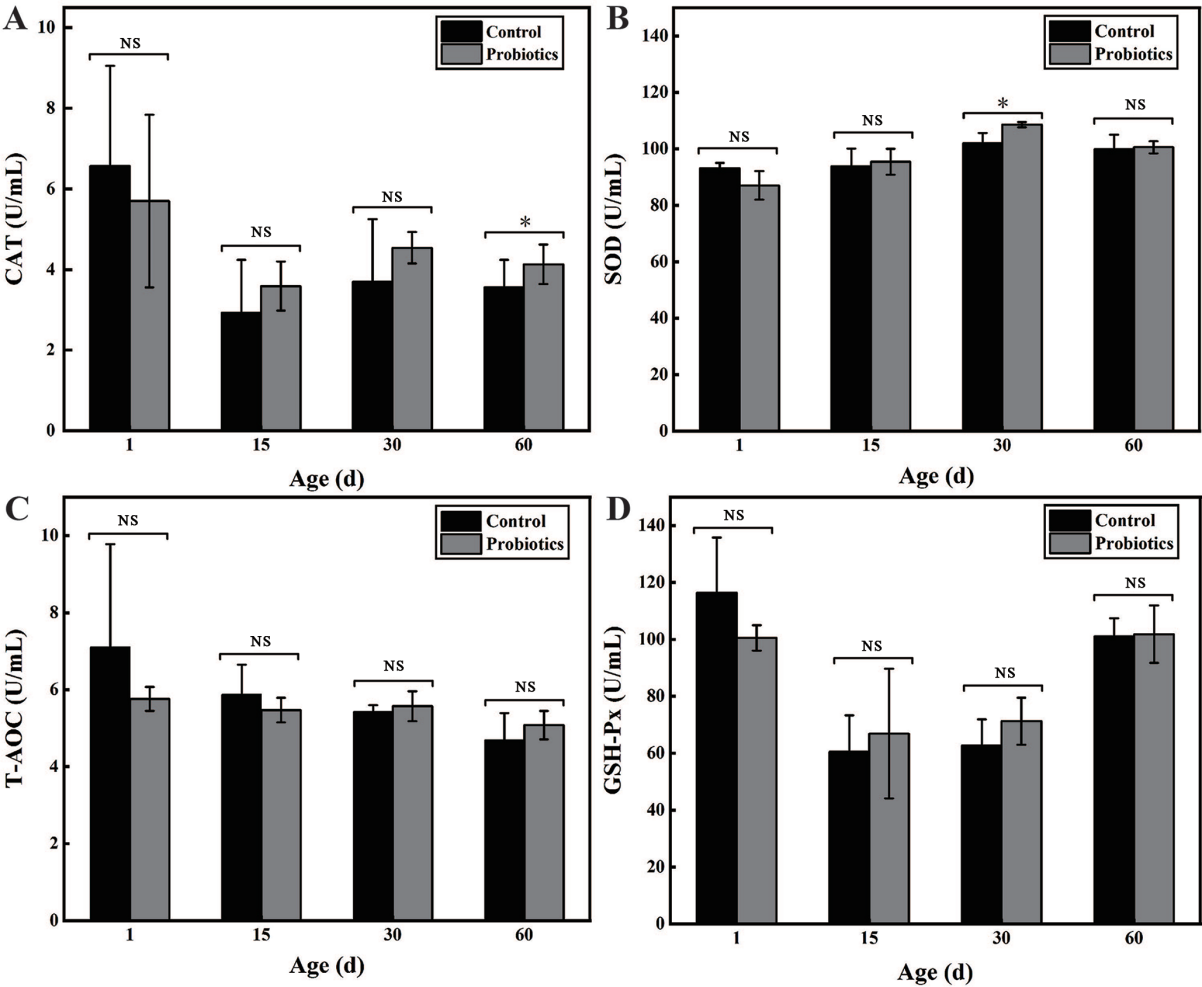
Effects of compound probiotics on the antioxidant ability of calves. (**A**) The activities of CAT. (**B**) The activities of SOD. (**C**) The activities of T-AOC. (**D**) The activities of GSH-Px. Note: “*” means “*P* < 0.05” and “NS” refers to “Non-significant.”

In order to explore the effect of feeding probiotics on the antioxidant indexes of calves, the activities of catalase (CAT), total antioxidant capacity (T-AOC), superoxide dismutase (SOD), and glutathione peroxidase (GSH-Px) in calf serum were further determined in this work. Results showed that continuous feeding of the compound probiotics could significantly increase the activities of CAT and SOD (*P* < 0.05; Fig. 3A and B), but the activities of T-AOC and GSH-Px did not change significantly (*P* > 0.05; Fig. 3C and D). For example, when the calves were fed with compound probiotics, the activities of CAT and SOD at 30 days were 4.54 ± 0.39 U/mL and 108.56 ± 0.92 U/mL, respectively, which were 18.50% and 6.0% higher than those of the control (3.7 ± 1.55 U/mL and 101.94 ± 3.68 U/mL). However, regarding the activities of T-AOC and GSH-Px, there was no significant difference between the calves fed with compound probiotics and those fed with normal saline. In conclusion, continuous feeding of the compound probiotics effectively improved the antioxidant capacity of calves, which indicated that probiotics can help reduce the oxidative damage caused by oxidative stress of calves. According to current reports, improving the antioxidant status of animals might accelerate the maturity of their immune system and keep them healthy, thus reducing the incidence of diseases (27). Therefore, intake of the compound probiotic might contribute to scavenging the free radicals in calves and improving the antioxidant capacity of calves (28). However, with the increase of calf age, the antioxidant capacities were gradually decreased, but the antioxidant capacity of calves fed with compound probiotics decreased slowly. In short, feeding the compound probiotics can effectively maintain higher antibody levels in calf serum, thus improving the disease resistance of newborn calves.

### Feeding compound probiotics improved the overall immunity of calves

**Fig 4:**
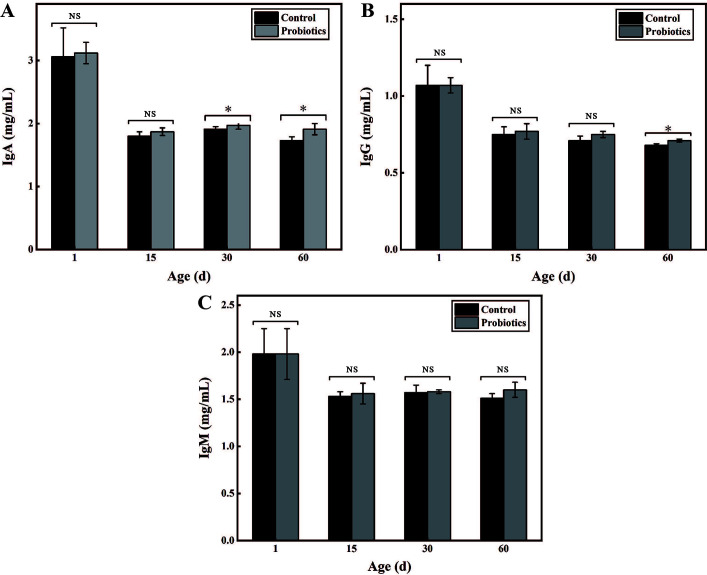
Effects of compound probiotics on the contents of antibodies in newborn calves. (**A**) The contents of IgA. (**B**) The contents of IgG. (**C**) The contents of IgM. Note: “*” means “*P* < 0.05” and “NS” refers to “Non-significant.”

To investigate the impact of compound probiotic on the immune function of calves, non-specific antibodies in calf serum, including IgA, IgG, and IgM, were further tested. As shown in Fig. 4, compared with the first day of birth, the levels of IgA, IgG, and IgM in calves showed a downward trend, regardless of whether they were fed with compound probiotics or normal saline, the largest decline of IgA reaching 76.88%. However, when the compound probiotics were fed for 30 days, the IgA level was significantly higher than that of the control (*P* < 0.05), which increased by 3.14% and 10.4% at 30 days and 60 days, respectively (Fig. 4A). Furthermore, when fed with the compound probiotics for 60 days, the IgG level of calves (0.71 ± 0.01 mg/mL) was still higher than that of the control (0.68 ± 0.01 mg/mL; Fig. 4B). In contrast, during the whole experiment period, no significant difference was observed in the IgM level between feeding compound probiotics and normal saline (*P* > 0.05; Fig. 4C). These results showed that feeding the compound probiotics could help to slow down the decline of IgA and IgG in calves and improve the non-specific immunity of calves during infancy (within 60 days), thus helping to decrease the diarrhea rate and improving the growth performance of newborn calves.

### Feeding compound probiotics contributes to regulating the gut microbiome of calves and promoting the development of intestinal function

To reveal the impact of feeding compound probiotic on the gut microbiome of calves, metagenomic sequencing and analysis of the calf feces were conducted. Metagenome sequencing data showed that the gut microbiome at the phylum level of newborn calves is extremely similar on the first day (Table S1), the dominant microbiomes are *Unclassified microorganisms*, *Firmicutes*, and *Proteobacteria*, respectively. With the growth and development of calves, the abundance of *Unclassified microorganisms* gradually decreased, while *Firmicutes* and *Bacteroidetes* gradually became the main flora in the intestines. When the calves were continuously fed with compound probiotics for 30 days, the abundance of *Candidatus Gottesmanbacteria* (up-regulated by 2.74-folds) and *Candidatus Ryanbacteria* (up-regulated by 2.28-folds) in the experimental calves was significantly higher than that in the control (Table 1). No significant difference (*P*＞0.05) was observed in the other phylum. However, when continuously feeding the compound probiotics for 60 days, the number of 26 phylum in the fecal microbiome of experimental calves was significantly up-regulated (e.g., *Candidatus Microgenomates* and *Candidatus Yanofskybacteria*; Table 2), and two phylum were significantly down-regulated (*Candidate Division WWE3* and *Fuso Bacteria*; Table 2). These evidences indicated that the calves continuously fed with compound probiotics for 60 days would significantly change the fecal microbiome at phylum level.

**TABLE 1:** Differences in the fecal microbiome at phylum level in 30-day-old calves

Taxon	Abundance of CK30-avg	Abundance of T30-avg	T30-vs-CK30 (log2fc)	*P*-value
*Candidatus Gottesmanbacteria*	6.15	174.2	1.45217	0.00446
*Candidatus Ryanbacteria*	0.65	10	1.18709	0.00072

**TABLE 2:** Differences in the fecal microbiome at phylum level in 60-day-old calves

Taxon	Abundance of CK60-avg	Abundance of T60-avg	T60-vs-CK60 (log2fc)	P-value
*Candidatus Microgenomates*	2.05	64.85	4.983	0.01004
*Candidatus Yanofskybacteria*	6.45	179.15	4.796	0.01132
*Candidatus Lloydbacteria*	0.6	15.5	4.691	0.00009
*Candidatus Levybacteria*	1	22.45	4.489	0.00293
*Candidatus Sumerlaeota*	1.75	24.95	3.834	0.00817
*Elusimicrobia*	23.2	305.95	3.721	0.00033
*Candidatus Zambryskibacteria*	2.35	29.9	3.669	0.00093
*Candidatus Bathyarchaeota*	10.3	104.35	3.341	0.03507
*Candidatus Daviesbacteria*	0.8	7.9	3.304	0.02232
*Tenericutes*	7457	65055.1	3.125	0.02064
*Candidatus Nomurabacteria*	9.75	80.55	3.046	0.03270
*Candidatus Yonathbacteria*	1.15	8.3	2.851	0.01348
*Parabasalia*	10.25	62.35	2.605	0.03796
*Acidobacteria*	145.55	793.7	2.447	0.03253
*Nitrospirae*	44.65	232.5	2.380	0.01990
*Candidatus Cryosericota*	8.2	38.7	2.239	0.00785
*Candidatus Parcubacteria*	34.65	141.95	2.034	0.00016
*candidate division Zixibacteria*	8	31.55	1.980	0.00068
*Thermodesulfobacteria*	13.85	52.6	1.925	0.00927
*Armatimonadetes*	18.2	67.7	1.895	0.04469
*Candidatus Melainabacteria*	2425.95	8652.75	1.835	0.00226
*Candidatus Bipolaricaulota*	1.65	5.65	1.776	0.03138
*Candidatus Aminicenantes*	9	30.05	1.739	0.04654
*Candidatus Saccharibacteria*	2631.6	7794.3	1.566	0.03028
*Chloroflexi*	612.2	1760.8	1.524	0.00183
*Euryarchaeota*	2347	5759.7	1.295	0.04678
*candidate division WWE3*	317.3	98.05	−1.694	0.01206
*Fusobacteria*	75005.6	4039.35	−4.215	0.01762

**Fig 5:**
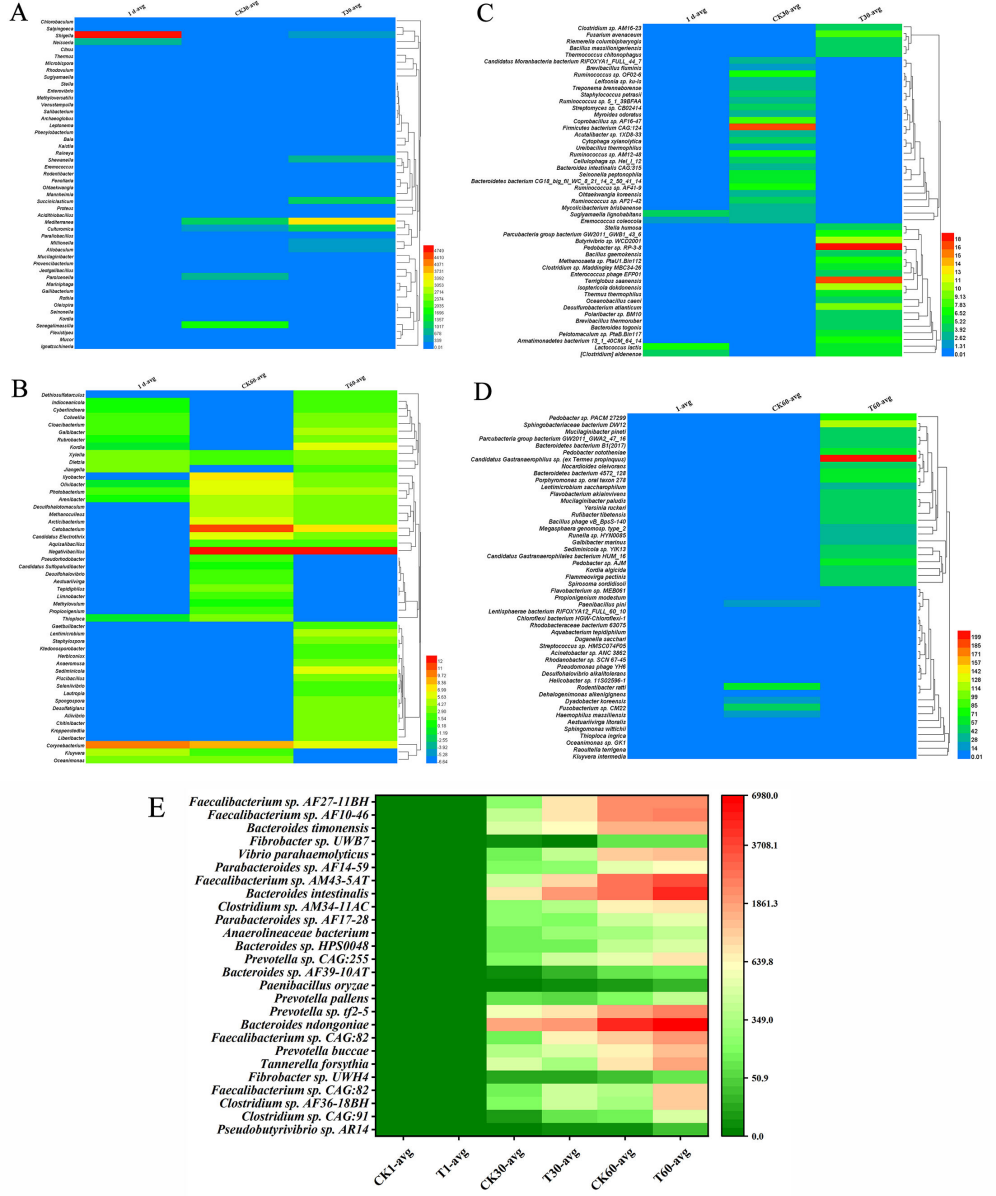
Effects of feeding compound probiotics on the fecal microbiome of calves. (**A**) Differences of the fecal microbiome at genus level in 30-day-old calves. (**B**) Differences of the fecal microbiome at genus level in 60-day-old calves. (**C**) Differences of the fecal microbiome at species level in 30-day-old calves. (**D**) Differences of the fecal microbiome at species level in 60-day-old calves. (**E**) Continuously up-regulated strains of calves when fed with the compound probiotics for 60 days.

With regard to the fecal microbiome at the genus level (Table S2), results indicated the calves fed with compound probiotics for 30 days would cause 32 genus of the fecal microbiome to be significantly up-regulated (e.g., *Archaeoglobus*, *Chlorobaculum*, and *Acidithiobacillus*), and 16 genus were significantly down-regulated (e.g., *Provencibacterium*, *Jeotgalibacillus*, and *Neisseria*; Fig. 5A; Table S3). When the calves were continuously fed with the compound probiotics for 60 days, the number of 97 genus in the fecal microbiome was significantly up-regulated (e.g., *Kordia*, *Sediminicola*, and *Rubrobacter*), and 21 genus in the fecal microbiome were significantly down-regulated (e.g., *Kluyvera*, *Oceanimonas*, and *Thioploca*; Fig. 5B; Table S4). Therefore, an increased abundance of some potentially beneficial bacteria (e.g., *Archaeoglobus*, and *Acidithiobacillus*) and a decreased abundance of some potentially harmful bacteria (e.g., *Neisseria *and *Kluyvera*) might contribute to maintaining a stable gut microbiome of calves (10, 29–33), which is conducive to improving intestinal health and reducing the incidence of diarrhea caused by microbiome disorder.

In terms of the fecal microbiome at species level (Table S5), the metagenome sequencing results demonstrated that calves fed with compound probiotics for 30 days would lead to the significant up-regulation in the abundance of 160 microbial species (e.g., *Lactococcus lactis*, *Brevibacillus thermoruber*, and *Bacillus massilionigeriensis*), and 85 microbial species were significantly down-regulated (e.g., *Escherichia sp. MOD1-EC5189*, *Mycolicibacterium brisbanense*, and *Treponema brennaborense*; Fig. 5C; Table S6). When the calves were continuously fed with compound probiotics for 60 days, the abundance of 481 microbial species was significantly up-regulated (e.g., *Candidatus Gastranaerophilales bacterium HUM_16*, *Ruminococcus sp. AM49-8*, and *Lactobacillus lindneri*), while 114 microbial species were significantly down-regulated (e.g., *Helicobacter pullorum*, *Tuberibacillus sp. Marseille-P3662*, and *Haemophilus massiliensis*; Fig. 5D; Table S7). Among the significantly up-regulated strains, some potential functional probiotics were found, such as *L. lactis*, *Bacillus massionigeriensis*, etc*.* Among them, *L. lactis* has progressed a long way since its discovery and initial use in dairy product fermentation, which is a typical probiotic and has been widely used in the food and medicine industry (34). *B. massilionigeriensis* is a strain that exists in healthy human intestines, which may be beneficial to maintain a stable gut microbiome *in vivo* (35). Regarding the significantly down-regulated strains, we found that some of them are considered potentially harmful strains, such as *Escherichia sp. MOD1-EC5189*, *Mycobacterium brisbane*, etc*.* Among them, *Escherichia sp*., it is generally known as the opportunistic pathogen, which will cause diseases including diarrhea, enteritis, and respiratory tract infection (36). *Mycolicibacterium* is usually a zoonotic infectious pathogen, which can cause tuberculosis, lung infection, complicated infection, and other diseases (37).

In addition, according to the species annotation of metagenome sequencing, it was found that intervention of the compound probiotics mediated continuous up-regulation of 26 strains within 60 days (Fig. 5E), among which four strains were significantly up-regulated compared with the control (*P* < 0.05, Table 3), including *Prevotella sp. CAG:255*, *Clostridium sp. AF36-18BH*, *Faecalibacterium sp. CAG:82*, and *Pseudobutyrivibrio sp. AR14*. For example, the calves fed with compound probiotics for 30 days increased the abundance of *Prevotella sp. CAG:255*, *Clostridium sp. AF36-18BH*, *Faecalibacterium sp. CAG:82*, and *Pseudobutyrivibrio sp. AR14* by 1.97-folds, 1.81-folds, 3.90-folds, and 4.07-folds, respectively. Moreover, when calves were continuously fed with the compound probiotics for 60 days, the abundances of the above four strains were further up-regulated by 1.31-folds, 3.28-folds, 1.88-folds, and 4.04-folds, respectively, indicating these four strains are very important in responding to the intervention of the compound probiotics. Regarding the down-regulated strains, none of them were found to be continuously significantly down-regulated within 60 days. However, we found that the abundance of *Yarrowia lipoytica* and *Frankia sp. CPI 1*P was significantly down-regulated to a very low level when fed with compound probiotics for 30 days, which could not be further reduced, so it was difficult to observe whether the strains were continuously down-regulated within 60 days. Therefore, with the growth and development of calves, the gut microbiome of healthy calves was maintained at a relatively stable level, thus effectively reducing the incidence of diarrhea, which is consistent with the above results.

**TABLE 3:** The continuous significantly up-regulated strains of calves mediated by the compound probiotics

Comparison	*Prevotella sp. CAG:255*	*Clostridium sp. AF36-18BH*	*Faecalibacterium sp. CAG:82*	*Pseudobutyrivibrio sp. AR14*
CK1-abundance	0.0001	0.0001	0.0001	0.0001
T1- abundance	0.0001	0.0001	0.0001	0.0001
CK30- abundance	221.95	229.1	169.25	3
T30- abundance	438.2	414.55	660.75	12.2
CK60- abundance	579.25	337.95	1090.2	9.1
T60- abundance	757.4	1106.8	2054.25	36.8
Log_2_fc of T30-vs-CK30	0.9814	0.8556	1.9649	2.0238
*P*-value of T30-vs-CK30	0.0056	0.0009	0.0002	0.0000
Log_2_fc of T60-vs-CK60	0.3869	1.7115	0.9140	2.0158
*P*-value of T60-vs-CK60	0.0065	0.0000	0.0159	0.0104

In a word, feeding calves with compound probiotics for 30 or 60 days would significantly change the fecal microbiome, increase the abundance of some beneficial microorganisms, and decrease the abundance of some potential harmful strains in calves, thus contributing to accelerating the construction of gut microbiome in calves.

### Feeding compound probiotics affected the nutritional metabolism of calves

**Fig 6:**
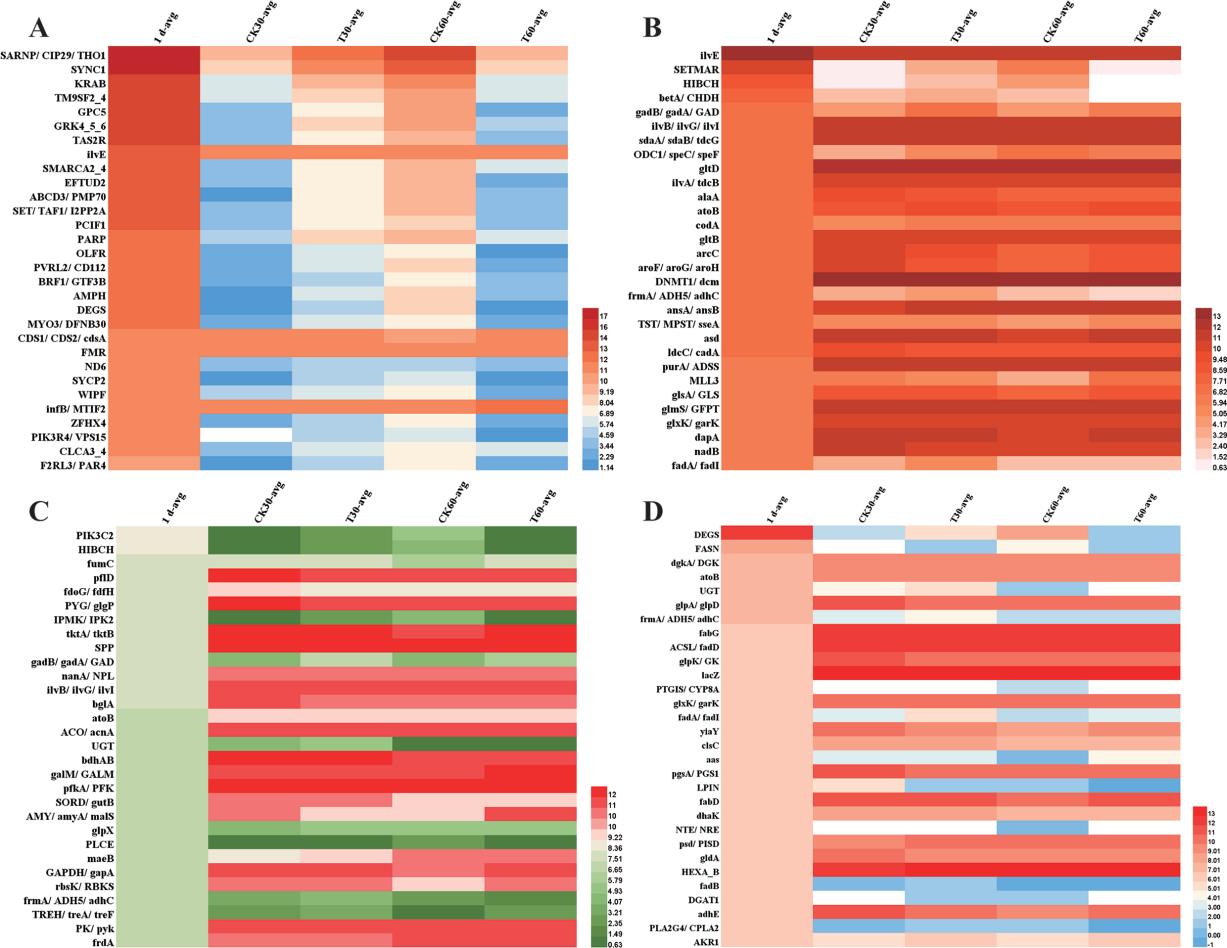
Effect of feeding compound probiotics on the nutritional metabolism of calves. (**A**) The expression abundance of Top 30 genes in the gut microbiome of caves. (**B**) Top 30 genes in the gut microbiome that involved in the regulation of amino acid metabolism. (**C**) Top 30 genes in the gut microbiome that involved in the regulation of carbohydrate metabolism. (**D**) Top 30 genes in the gut microbiome that involved in the regulation of lipid metabolism.

To further evaluate the effects of feeding compound probiotics on the nutritional metabolism of calves, the main differentially expressed genes involved in metabolic regulation were analyzed according to the results of metagenomic sequencing. Regarding the Top 30 genes involved in the regulation of amino acid metabolism in calves, continuous feeding of compound probiotics for 30 days significantly decreased the expression abundance of *aroF*, *aroG*, and *aroH* genes and increased the expression abundance of *fadA* and *fadI* genes (*P* < 0.05; Fig. 6B; Table S8). Among them, *aroF*, *aroG*, and *aroH* are involved in catalyzing the production of initial substrates for aromatic amino acid biosynthesis and participate in the biosynthesis of phenylalanine, tyrosine, and tryptophan (38). The *fadA* gene participates in the degradation of long-chain fatty acids, increasing the intracellular supply of acetyl-CoA, NADH, and ATP, improving the metabolic flux of the tricarboxylic acid (TCA) cycle, and regulating the amino acid synthesis of TCA derivatives (39). These results suggest that feeding probiotics might reduce the intake of phenylalanine, tyrosine, and tryptophan in the calf intestine and improve the metabolism and absorption of fatty acids, which could be beneficial to accelerate the growth and development of calves.

In terms of the Top 30 genes involved in the regulation of carbohydrate metabolism, feeding compound probiotics or normal saline did not affect their expression abundance, suggesting the basic carbohydrate metabolism and nutrient absorption of newborn calves were relatively stable (Fig. 6C; Table S8). However, with the growth of calves, the abundance of *PYG*, *glgP*, *tktA*, *tktB*, and *bglA* genes increased significantly (*P* < 0.05), while the abundance of *PIK3C2*, *HIBCH*, *IPMK*, *IPK2*, *UGT,* and *PLCE* genes decreased significantly (*P* < 0.05). Among the up-regulated genes, *pfkA* and *PFK* genes are involved in catalyzing the conversion of D-fructose 6-phosphate to fructose 1,6-diphosphate via ATP phosphorylation and are involved in the first step of glycolysis. The *PYK* gene is involved in encoding the enzyme that catalyzes the last step of glycolysis, which is the conversion of phosphoenolpyruvate into pyruvate (40). The down-regulated genes (e.g., *IPMK* and *IPK2*) are mainly involved in regulating the metabolism of isopentenyl diphosphate and phosphoinositol. These results suggested that in the early growth of calves, proper supplementation of the intermediate metabolites of carbohydrates may contribute to accelerating their growth and development, such as fructose-1,6-diphosphate and pyruvate.

In the case of lipid metabolism, the expression abundance of most Top 30 genes showed a significant fluctuation trend within 60 days when the calves were fed with normal saline, such as *glpD*, *pgsA*, *PGS1*, *fadB*, *fadA*, etc. (Fig. 6D; Table S8). These genes are mainly involved in glycerol degradation, acidic phospholipid synthesis, and degradation of long-chain fatty acids and are mainly responsible for regulating glycerol phospholipid metabolism and fatty acid biosynthesis (41). However, when the calves were fed with compound probiotics, the expression abundance of these genes was relatively stable within 60 days, suggesting that the compound probiotics contributed to maintaining the steady state of lipid metabolism, ensuring the nutritional metabolism and absorption of fatty acids, and reducing the diarrhea caused by lipid metabolism disorder.

## DISCUSSIONS

The gut microbiome plays an important role in regulating the intestinal development and immune function of mammals (42, 43). However, the species and abundance of gut microbiome usually show a dynamic changing rule and are easily influenced by various factors, such as diet, antibiotic intervention, and invasion of pathogenic microorganisms. When the intestinal beneficial bacteria in the host were deceased, and the pathogenic bacteria or conditional pathogenic bacteria were increased, many intestinal diseases, such as intestinal dysfunction, diarrhea, gastroenteritis, and other diseases, would occur (44–46). Moreover, the gut microbiome of animals can not only directly affect the immune function and energy metabolism of the host but also regulate the nutritional and metabolic activities by producing various metabolites (e.g., lactate and fatty acid). Furthermore, some metabolites, such as short-chain fatty acids, are secreted by some representative strains of *Faecalibacterium* (e.g., *F. longum* and *F. prausnitzii*) and have anti-inflammatory effects by blocking the production of NF-kB and IL-8 (44). Studies also found that some specific species of *Bacteroides* (e.g., *Bacteroides fragilis*) contributed to preventing *Clostridium difficile* infection (47, 48), such as stimulating paneth cell protein to produce antibacterial peptides and preventing pathogens colonization in the gut of host (43). Therefore, the regulation of the gut microbiome is of great significance to the health and growth of animals. In this work, we screened four typical probiotics (including *L. plantarum*, *E. faecium*, *S. cerevisiae*, and *B. licheniformis*) from the fermented feed of healthy cows and developed the compound probiotics, which contributed to accelerating the construction of gut microbiome and immunologic function in newborn calves.

According to the metagenome sequencing data, it was found that feeding the compound probiotics could significantly change the fecal microbiome of calves. For example, compared to the control, the calves continuously fed with the compound probiotics for 60 days would lead to significant changes in the abundance of 595 strains, including 481 up-regulated strains (e.g., *Candidatus Gastranaerophilales bacterium HUM_16*, *Ruminococcus sp. AM49-8*, and *Lactobacillus lindneri*) and 114 down-regulated strains (e.g., *H. pullorum*, *Tuberibacillus sp. Marseille-P3662*, and *H. massiliensis*). However, the intaked *S. cerevisiae* was not found in the calf feces, but the intaked *Enterococcus faecalis*, *L. plantarum*, and *B. licheniformis* were detected. The abundance of *Enterococcus faecalis* and *L. plantarum* in the experimental calves was not significantly different compared to the control (the calves fed with normal saline). These results suggested that the intaked compound probiotics might stay in the gut of calves via competitive occupation or have been consumed or declined as nutrients in the intestine. However, the specific process and mechanism of change are still unclear and need further research. In short, although some probiotics were not detected (e.g., *S. cerevisiae*) or the abundance of other strains (e.g., *Enterococcus faecalis* and *L. plantarum*) was not significantly changed in the calf feces, continuous feeding of the compound probiotics for 30 or 60 days significantly changed the fecal microbiome of calves, among which including the continuous significantly up-regulated strains (e.g., *Prevotella sp. CAG:255* and *Pseudobutyrivibrio sp. AR14*). Combined with the above changes of diarrhea rate, serum antioxidant activity, and serum antibody level in calves, it was concluded that feeding the compound probiotics effectively regulated the gut microbiome, reduced the diarrhea rate, and improved the overall disease resistance of newborn calves. This study provides a new technical scheme for the production and application of compound probiotics in mammals.

For most animals, the intake of exogenous microorganisms usually causes an immune response, which may lead to oxidative stress and the changes of non-specific antibodies in animals. As a functional additive commonly used in animals, probiotics could reduce the incidence of diarrhea by regulating the structure of the gut microbiome (49) and could improve the level of non-specific antibodies in animals by stimulating immune response, thus contributing to enhancing the comprehensive disease resistance of animals (50). Regarding the antioxidant capacity and non-specific immunity of newborn calves, we found that feeding compound probiotics effectively improved the antioxidant indexes (e.g., SOD and CAT) of calves in this work. According to the current reports, improving the antioxidant status of animals might accelerate the maturity of their immune system and maintain a healthy state, thereby reducing the incidence of diseases (27), suggesting that intake of the compound probiotic contributed to scavenging the free radicals in calves and improving their antioxidant capacity (28). However, with the increase of calf age, their antioxidant capacities were found gradually decreased, but it is slower for the calves fed with compound probiotics. In short, intake of the compound probiotics effectively maintained the higher levels of antioxidant capacities in calf serum, thus improving the disease resistance of newborn calves. In terms of non-specific immunity, results showed that intake of the compound probiotics is helpful to slow down the decay rate of the non-specific antibodies (e.g., IgA and IgG). For most animals, immunoglobulin is a family of bioactive globular proteins with antibacterial and protective properties, among which IgM, IgA, and IgG are important indicators of immune function. In general, IgM is the first antibody produced in the early stage of the immune response and is the first line of defense against pathogens (51). IgA is the main antibody of exocellular secretions and plays a key role in immune protection (52). IgG plays an important role in the systemic immune response and is the main antibody after ingesting probiotic protein. Therefore, our study indicated that probiotics could act as immunomodulators, which can interact with the gut microbiome, epithelial cells, and immune cells to stimulate the immune function of newborn calves, thereby improving the defense ability of host against pathogenic microorganisms (53, 54).

In summary, the unstable gut microbiome and incomplete development of the intestinal function of newborn calves are the important reasons for the high incidence of early diarrhea. Feeding probiotics contributed to accelerating the establishment of gut microbiome and immune barrier, thus resisting the invasion of foreign pathogens and promoting the growth performance of calves. This study provides new insights into the regulation mechanism of probiotics on mammalian gut microbiome.

## MATERIALS AND METHODS

### Materials

One thousand grams of the fermented feed was collected from a dairy farm in Laibin, Guangxi, China (109.10°E, 23.77°N). Glucose, peptone, yeast extract, agar powder, NaCl, K_2_HPO_4_, and KH_2_PO_4_ were all of analytical grade and purchased from Beijing Solaibao Science and Technology Co., Ltd. (Beijing, China). GSH-Px assay kit, SOD assay kit, CAT assay kit, and T-AOC assay kit were purchased from Nanjing Jiancheng Bioengineering Institute (Nanjing, China). The bovine immunoglobulin A (IgA) kit, bovine immunoglobulin G (IgG) kit, and bovine immunoglobulin M (IgM) kit were purchased from Shanghai Enzyme-linked Biotechnology Co., Ltd. (Shanghai, China).

### Isolation and identification of strains

Microorganisms in the fermented feed were isolated using Luria-Bertani (LB) medium (1% NaCl, 1% peptone, and 0.5% yeast extract) and deMan Rogosa Sharpe (MRS) medium (1% peptone, 1% beef extract, 0.5% yeast extract, 2% glucose, 0.01% MgSO4, 0.5% sodium acetate, 0.2% ammonium citrate, 0.2% K_2_HPO_4_, 0.005% MnSO_4_, 0.1% Tween 80, and pH 6.2 ± 0.2). Weigh 10 g of fermented feed and shake well in 90 mL of sterile water. After appropriate dilutions, the samples were coated on LB and MRS media. After incubation at 37°C for 24–48 h, the single colonies of strains were obtained. Identification of the strains was performed through 16S rDNA sequencing or internal transcribed spacer (ITS) sequencing by Sangon Biotech (Shanghai) Co., Ltd. (Shanghai, China). The primer sequences for amplifying of bacteria V3-V4 region are F: 5′-AGTTTGATCMTGGCTCAG-3′ and R: 5′-GGTTACCTTGTTACGACTT-3′. The primer sequences for amplifying fungal ITS1F–ITS2R region are F: 5′- TCCGTAGGTGAACCTGCGG-3′ and R: 5′-TCCTCCGCTTATTGATATGC-3′. The sequenced 16S rRNA genes were compared with BLAST (https://blast.ncbi.nlm.nih.gov/Blast.cgi) using the NCBI blast similarity search tool. The phylogenetic analysis of our sequence with the closely related sequence from the blast results was performed followed by multiple sequence alignment (55).

### Biological antagonistic properties of the strains

After 16S rDNA sequencing or ITS sequencing, four strains of probiotics were obtained (Supplementary Material 1: Fig.S1 through S4), which were confirmed as safe probiotics according to the *Guidelines for the Identification and Safety Evaluation of Direct Feeding Microorganisms and Production Strains of Fermented Products* issued in China. In order to explore whether these probiotics can coexist, biological antagonistic experiments on them were conducted. First, 50 µL suspension of the *B. licheniformis*, *Enterococcus*, and *S. cerevisiae* were, respectively, coated on the independent media. Subsequently, 50 µL suspension of the *Enterococcus*, *L. plantarum*, and *S. cerevisiae* was inoculated on the *B. licheniformis* plate; 50 µL suspension of the *L. plantarum* and *S. cerevisiae* was inoculated on the *E. faecium* plate, and 50 µL suspension of the *L. plantarum* was inoculated on the *S. cerevisiae* plate. The experiments were carried out at 37°C for 48 h.

### Preparation of compound probiotics

The above four probiotics were all cultured to the OD600 between 0.8 and 1.0 and were then mixed according to equal volume (vol/vol) to obtain the compound probiotics. Subsequently, the compound probiotics were cultured at 37°C for 48 h to increase the viable counts and ensure that it meets the requirements of subsequent experiments. Total viable counts of the compound probiotics were detected via the plate counting method.

### Grouping and feeding scheme of the newborn calves

Fourteen healthy newborn calves were randomly divided into the control group and the experimental group. The average birth weight of calves in the control group was 36.8 kg, while that in the experimental group was 36.7 kg. Each calf in the control group was fed with 40 mL of normal saline per day, while each calf in the experimental group was fed with 40 mL of compound probiotics per day (the compound probiotics were shaken and divided into 40 mL, which were added directly into the foods for calves before feeding).

### Sample collection and metagenome sequencing

Three calves were randomly selected from the control or the experimental group. Calf blood samples were collected through jugular vein with blood anticoagulation tube on days 1, 15, 30, and 60, respectively. The serum was then collected by centrifugation at 4,000 × g for 10 minutes and then quickly stored at −20°C for subsequent detection. The fresh feces from each selected calf were collected via sterile plastic bag, which were then pretreated and extracted the total DNA using Hipure Stool DNA Mini Kit (Guangzhou Magen Biotechnology Co., Ltd., Guangzhou, China). The DNA samples were subjected to metagenome sequencing by GENEWIZ Biotechnology Co., Ltd (Suzhou, China) according to the method of Lin et al. (56).

### Detection of the physiological and biochemical indexes

The diarrhea of calves was recorded every day. Where the fecal score is 0 = normal consistency; 1 = semiformed or pasty; 2 = loose but enough consistency to remain on bedding; 3 = watery feces that sift through bedding material (19). The diarrhea rate was calculated by Eq (1).


(1) $$Diarrhea\;rate\;(\%)=\frac{total\;number\;of\;diarrhea}{total\;number\;of\;heads\;\times \;experimental\;days}\;\times \;100$$

The birth weight of calves was recorded, and the final weight of calves was weighed on an empty stomach on days 15, 30, and 60, respectively. The average daily gain of calves was calculated by Eq. (2).


(2) $$Average\;daily\;gain\;(g/d)=\frac{final\;weight\;-\;birth\;weight}{days}$$

### Detection of the antioxidant indexes and immune indexes

The activities of GSH-Px, SOD, CAT, and T-AOC in calf serum were detected by the GSH-Px assay kit, SOD assay kit, CAT assay kit, and T-AOC assay kit, respectively. The contents of IgA, IgG, and IgM in calf serum were detected by IgA test kit, IgG test kit, and IgM test kit, respectively. The operation steps were in accordance with the instructions.

### Data analysis

Metagenome sequencing results were subjected to base calling using software Bcl2fastq (v2.17.1.14) to obtain the raw sequencing data. Cutadapt software (v1.9.1) was then used to remove the adapters and low-quality sequences from raw sequencing data. MEGAHIT (v1.1.3) software and Prodigal (v3.02) software were employed to perform assembly analysis and gene prediction for the metagenomic data, respectively. The MMseq2 sequence clustering software was utilized to further perform redundant removal and clustering of the gene sequences across all samples. Based on the predicted protein sequences of the coding genes, BLAST software (v2.2.31+) was used to compare them with protein sequences in databases to obtain annotation results for each gene. In addition, Diamond software was employed to compare the unigene sequences with NR database, and the taxonomic annotation information corresponding to each sequence in the non-redundant (NR) database was used to obtain species annotation results for each sequence, thus evaluating the relative abundance differences of the gut microbiome at phylum, genus, and species level. When the fold change (fc) of relative abundance differences meets the requirement of |Log_2_fc| ≥ 1, false discovery rate (FDR) value＜0.01, and *P*-value ＜0.05 are defined as significant difference. Statistical analysis of the experimental data is carried out by software SPSS 26. Origin 2021 software was used to draw graphics. The gene sequence was matched with the NCBI database, and the best match was chosen as the annotation result. The metabolic pathway and biological functions of the functional genes are annotated according to the KEGG or NCBI databases.

## Data Availability

The metagenome sequencing raw data were deposited to the NCBI under the accession number PRJNA924947.

## SUPPLEMENTAL MATERIAL

Supplemental figures spectrum.01909-23-s0001.docx Figures S1 to S4.

Graphical abstract spectrum.01909-23-s0002.tif Graphical abstract.

Table S1 spectrum.01909-23-s0003.xlsx The abundance of fecal microbiome at phylum level of calves.

Table S2 spectrum.01909-23-s0004.xlsx The abundance of fecal microbiome at genus level of calves.

Table S3 spectrum.01909-23-s0005.xlsx Differences of the fecal microbiome at genus level in 30-day-old calves.

Table S4 spectrum.01909-23-s0006.xlsx Differences of the fecal microbiome at genus level in 60-day-old calves.

Table S5 spectrum.01909-23-s0007.xlsx The abundance of fecal microbiome at species level of calves.

Table S6 spectrum.01909-23-s0008.xlsx Differences of the fecal microbiome at species level in 30-day-old calves.

Table S7 spectrum.01909-23-s0009.xlsx Differences of the fecal microbiome at species level in 60-day-old calves.

Table S8 spectrum.01909-23-s0010.xlsx (A) Abundance of top 30 genes of the fecal microbiome. (B) Top 30 genes of the fecal microbiome involved in the regulation of amino acid metabolism. (C) Top 30 genes of the fecal microbiome involved in the regulation of carbohydrate metabolism. (D) Top 30 genes of the fecal microbiome involved in the regulation of lipid metabolism.

ASM does not own the copyrights to Supplemental Material that may be linked to, or accessed through, an article. The authors have granted ASM a non-exclusive, world-wide license to publish the Supplemental Material files. Please contact the corresponding author directly for reuse.
