# Supplementary material for: Intake of compound probiotics accelerates the construction of immune function and gut microbiome in Holstein calves
Source: Microbiol Spectr. 2024 Apr 23;12(6):e01909-23. doi: 10.1128/spectrum.01909-23 (PMC11237676; doi:10.1128/spectrum.01909-23)

**Supplementary material 1. Identification results of the strains**

**Fig. S1.** *Lactobacillus plantarum*. (A) Agar plate of milk sample. (B) Plate marking of *Lactobacillus plantarum*. (C) Microscopic observation of *Lactobacillus plantarum*. (D) Evolutionary tree of *Lactobacillus plantarum*.


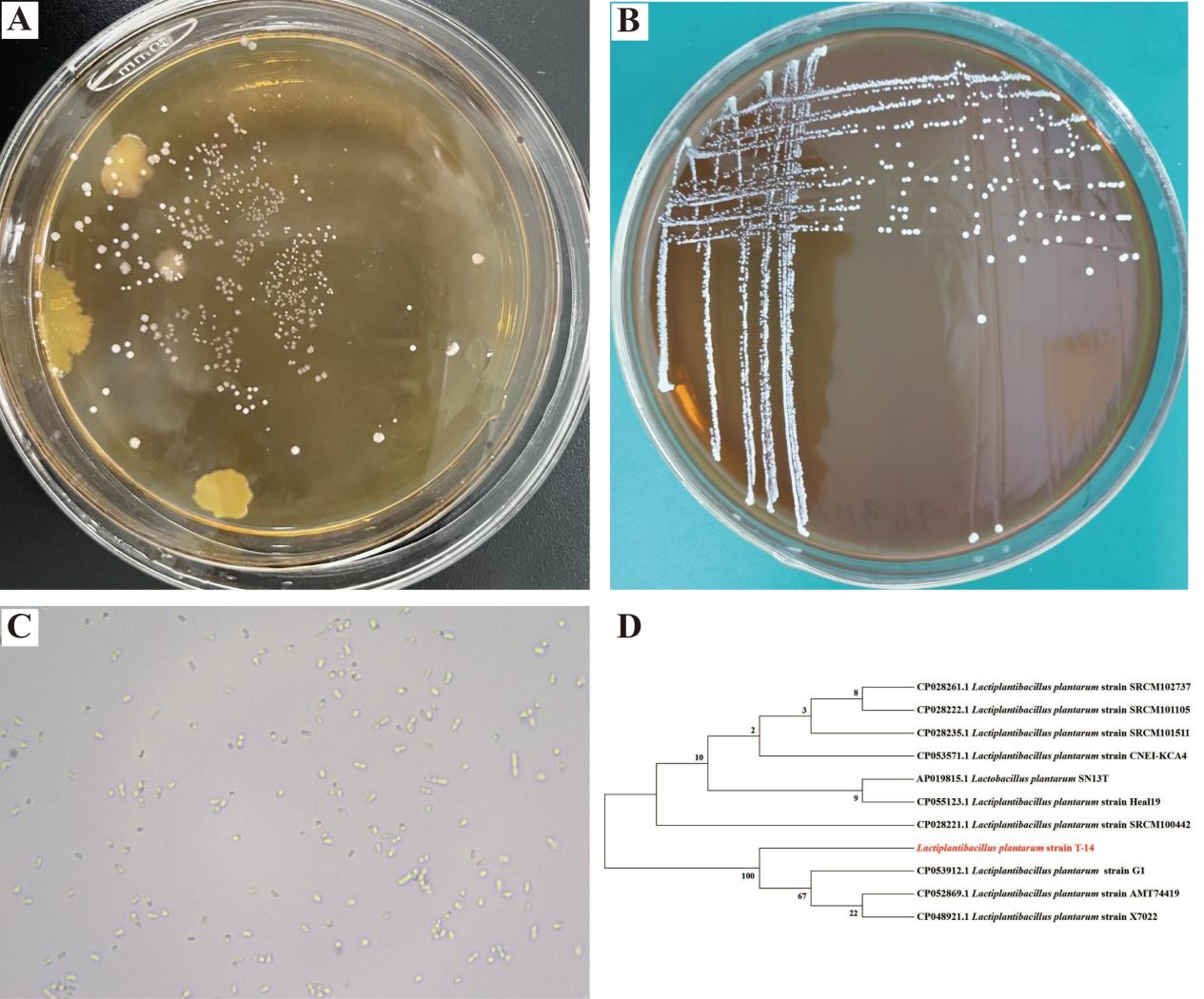


**Fig. S2.** *Enterococcus faecium*. (A) Agar plate of milk sample. (B) Plate marking of *Enterococcus faecium*. (C) Microscopic observation of *Enterococcus faecium*. (D) Evolutionary tree of *Enterococcus faecium*.


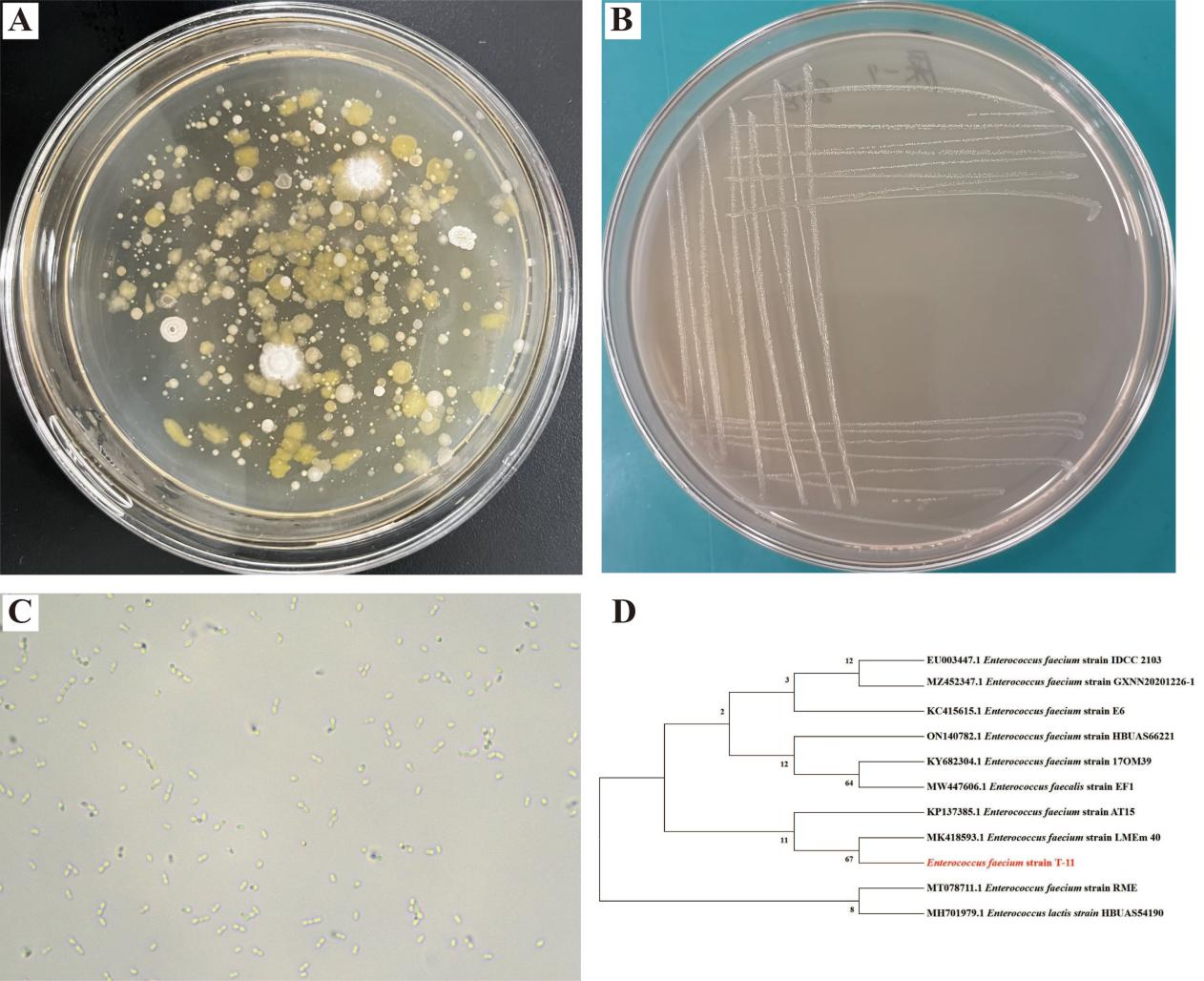


**Fig. S3.** *Saccharomyces cerevisiae*. (A) Agar plate of milk sample. (B) Plate marking of *Saccharomyces cerevisiae*. (C) Microscopic observation of *Saccharomyces cerevisiae*. (D) Evolutionary tree of *Saccharomyces cerevisiae*.


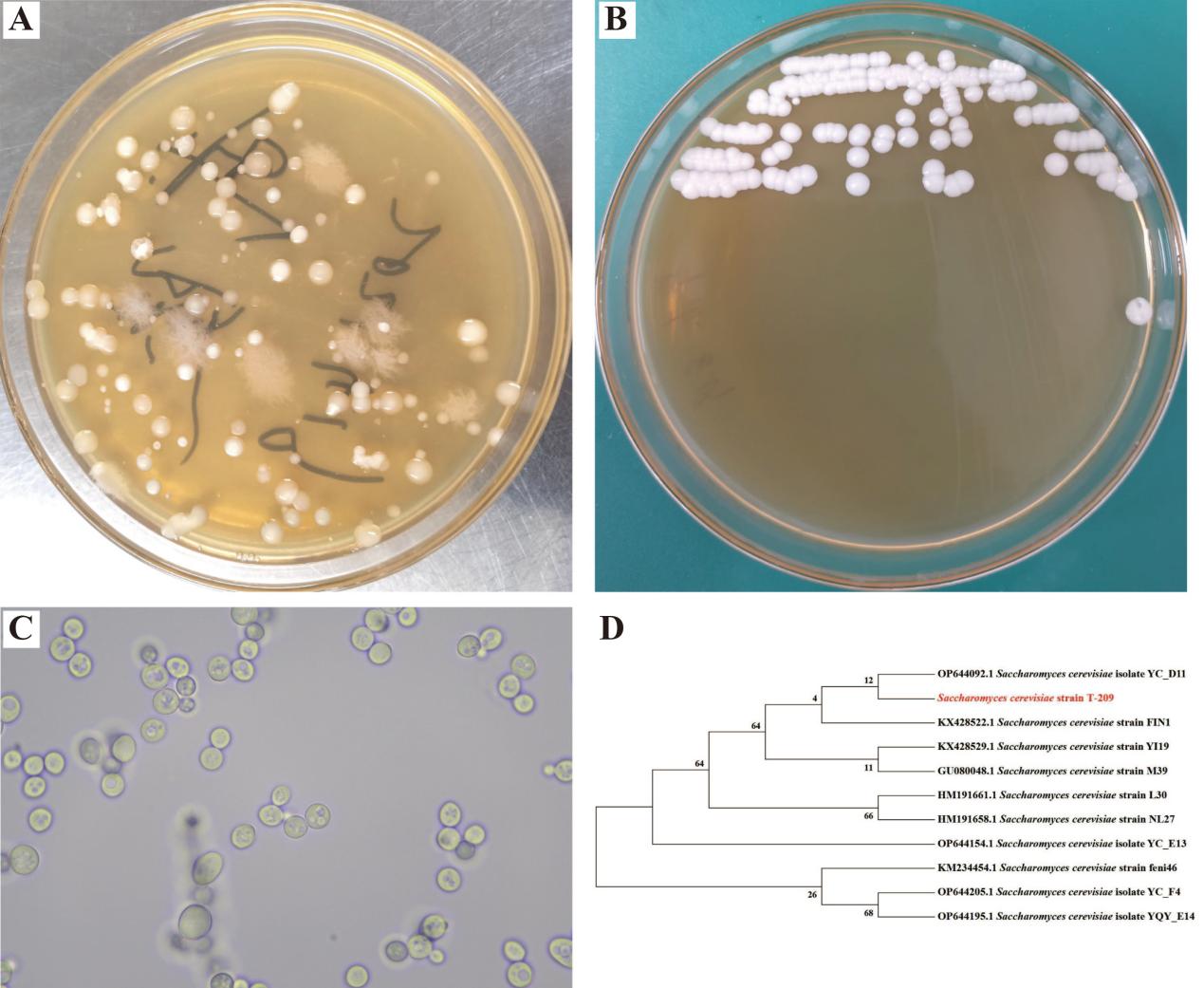


**Fig. S4.** *Bacillus licheniformis*. (A) Agar plate of milk sample. (B) Plate marking of *Bacillus licheniformis e*. (C) Microscopic observation of *Bacillus licheniformis e*. (D) Evolutionary tree of *Bacillus licheniformis*.


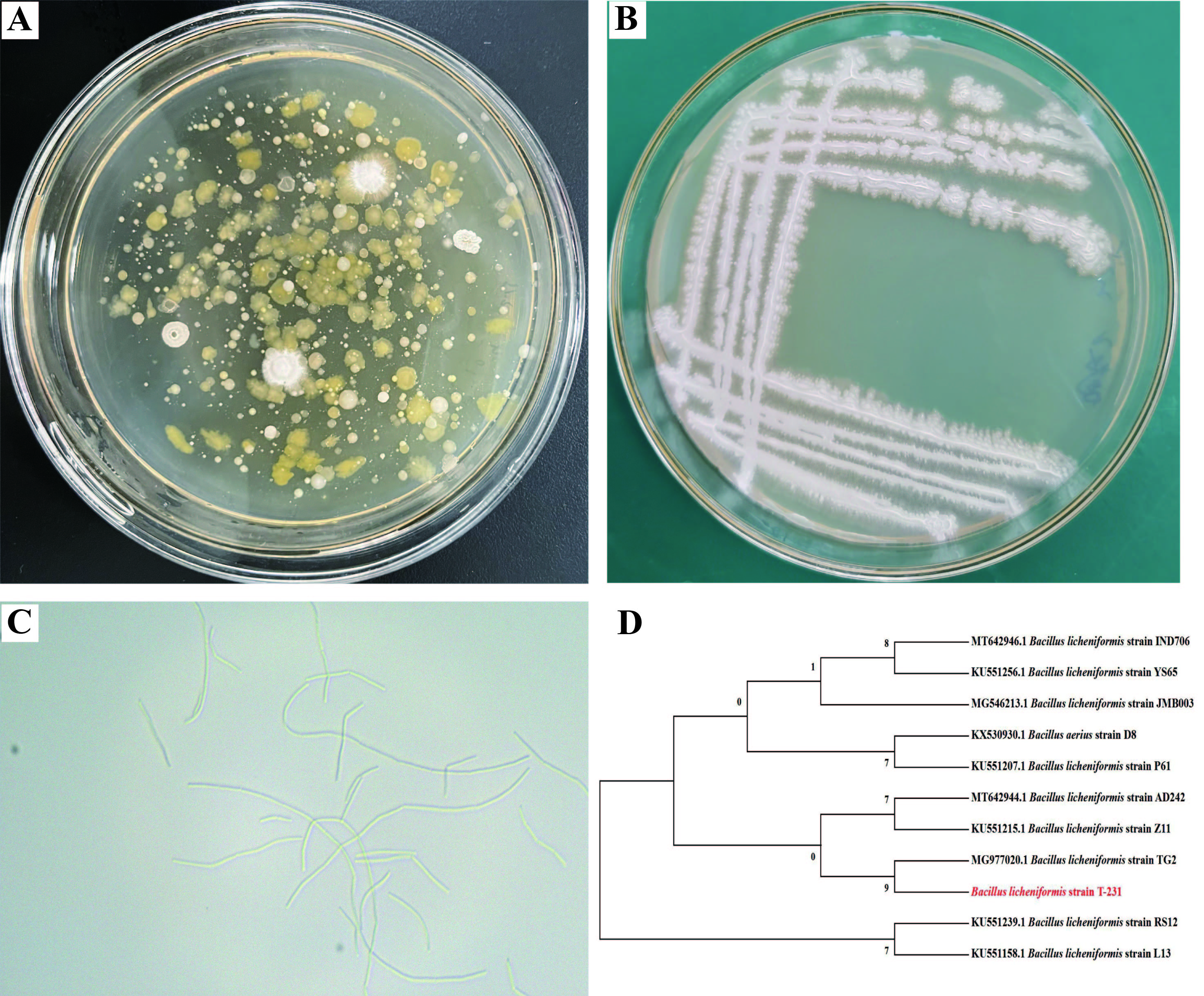

Supplement: Supplemental figures — Figures S1 to S4. [file spectrum.01909-23-s0001.docx]
