# Supplementary figures and images for: Intake of compound probiotics accelerates the construction of immune function and gut microbiome in Holstein calves
Source: Microbiol Spectr. 2024 Apr 23;12(6):e01909-23. doi: 10.1128/spectrum.01909-23 (PMC11237676; doi:10.1128/spectrum.01909-23)

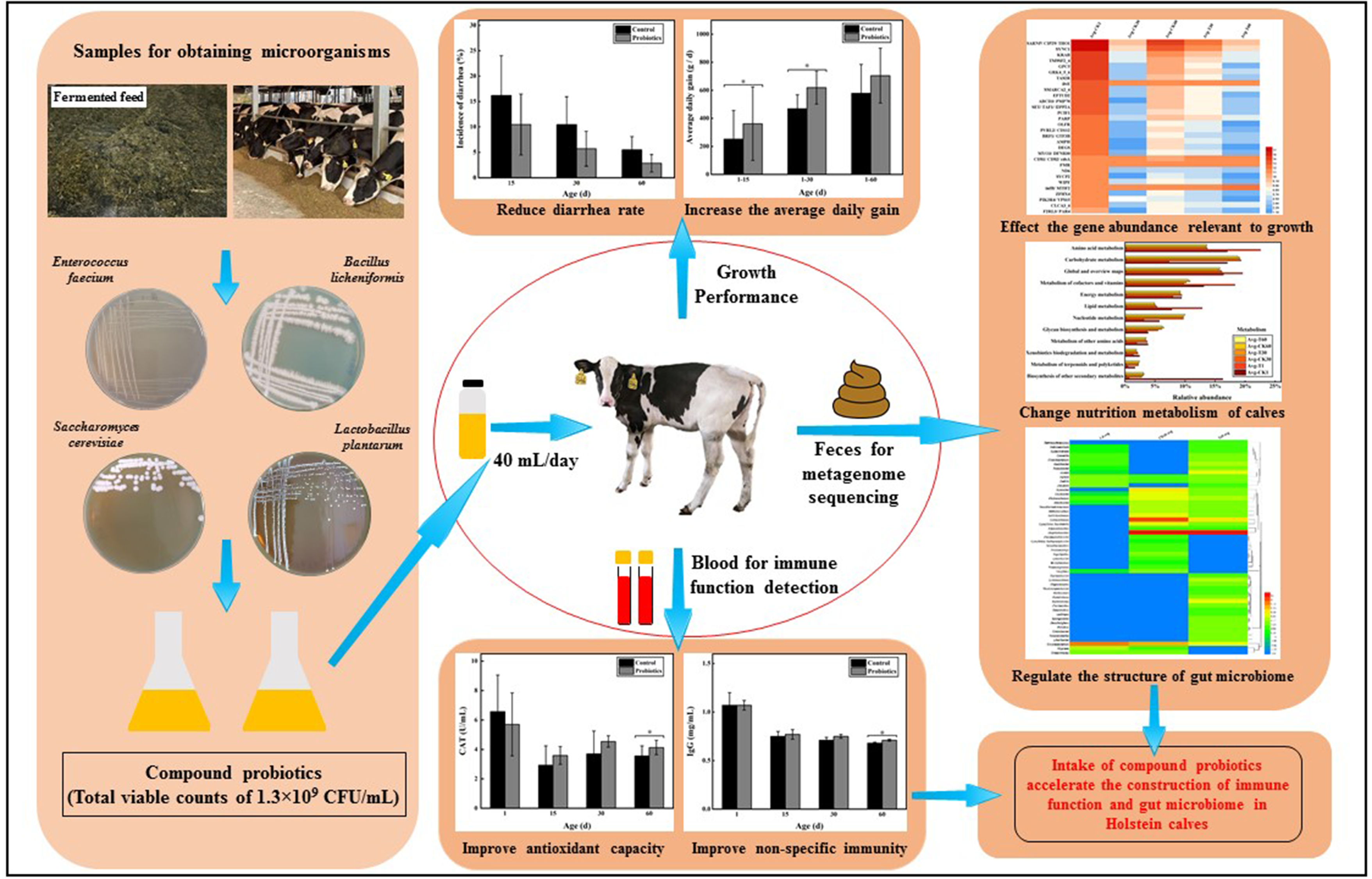

Supplement: Graphical abstract [file spectrum.01909-23-s0002.tif]
